# Early Postnatal Exposure to Ultrafine Particulate Matter Air Pollution: Persistent Ventriculomegaly, Neurochemical Disruption, and Glial Activation Preferentially in Male Mice

**DOI:** 10.1289/ehp.1307984

**Published:** 2014-06-05

**Authors:** Joshua L. Allen, Xiufang Liu, Sean Pelkowski, Brian Palmer, Katherine Conrad, Günter Oberdörster, Douglas Weston, Margot Mayer-Pröschel, Deborah A. Cory-Slechta

**Affiliations:** 1 Department of Environmental Medicine, and; 2 Department of Biomedical Genetics, University of Rochester School of Medicine, Rochester, New York, USA

## Abstract

Background: Air pollution has been associated with adverse neurological and behavioral health effects in children and adults. Recent studies link air pollutant exposure to adverse neurodevelopmental outcomes, including increased risk for autism, cognitive decline, ischemic stroke, schizophrenia, and depression.

Objectives: We sought to investigate the mechanism(s) by which exposure to ultrafine concentrated ambient particles (CAPs) adversely influences central nervous system (CNS) development.

Methods: We exposed C57BL6/J mice to ultrafine (< 100 nm) CAPs using the Harvard University Concentrated Ambient Particle System or to filtered air on postnatal days (PNDs) 4–7 and 10–13, and the animals were euthanized either 24 hr or 40 days after cessation of exposure. Another group of males was exposed at PND270, and lateral ventricle area, glial activation, CNS cytokines, and monoamine and amino acid neurotransmitters were quantified.

Results: We observed ventriculomegaly (i.e., lateral ventricle dilation) preferentially in male mice exposed to CAPs, and it persisted through young adulthood. In addition, CAPs-exposed males generally showed decreases in developmentally important CNS cytokines, whereas in CAPs-exposed females, we observed a neuroinflammatory response as indicated by increases in CNS cytokines. We also saw changes in CNS neurotransmitters and glial activation across multiple brain regions in a sex-dependent manner and increased hippocampal glutamate in CAPs-exposed males.

Conclusions: We observed brain region– and sex-dependent alterations in cytokines and neurotransmitters in both male and female CAPs-exposed mice. Lateral ventricle dilation (i.e., ventriculomegaly) was observed only in CAPs-exposed male mice. Ventriculomegaly is a neuropathology that has been associated with poor neurodevelopmental outcome, autism, and schizophrenia. Our findings suggest alteration of developmentally important neurochemicals and lateral ventricle dilation may be mechanistically related to observations linking ambient air pollutant exposure and adverse neurological/neurodevelopmental outcomes in humans.

Citation: Allen JL, Liu X, Pelkowski S, Palmer B, Conrad K, Oberdörster G, Weston D, Mayer-Pröschel M, Cory-Slechta DA. 2014. Early postnatal exposure to ultrafine particulate matter air pollution: persistent ventriculomegaly, neurochemical disruption, and glial activation preferentially in male mice. Environ Health Perspect 122:939–945; http://dx.doi.org/10.1289/ehp.1307984

## Introduction

Air pollution has been associated with adverse neurological and behavioral health effects in children and adults. Recent epidemiological studies have reported associations between exposure to air pollutants and increased risk for autism (Becerra et al. 2013; Volk et al. 2011, 2013), cognitive decline (Power et al. 2011; Weuve et al. 2012), ischemic stroke (Lisabeth et al. 2008; Wellenius et al. 2012), schizophrenia (Pedersen et al. 2004), and depression (Lim et al. 2012). Exposures—in particular to ultrafine ambient particulate matter (UFP; < 100 nm in aerodynamic diameter), identified as potentially the most toxic constituent of air pollution (Oberdörster 2000)—are pervasive and ubiquitous. Increases in neuroinflammation, oxidative stress, and glial activation have been identified as putative mechanisms by which air pollution exposures may impair central nervous system (CNS) function in adults (Block and Calderón-Garcidueñas 2009), but such exposures in the context of early brain development—a time frame considered crucial to causation of autism, schizophrenia, and cognitive development—remain largely unexplored. Given the potential public health importance of the reported epidemiological associations, it is imperative that the biological plausibility of such early developmental exposures to produce CNS dysfunction and disease be examined. Thus, we hypothesized that exposure of mice to UFP during early postnatal development—a period of rapid brain growth and differentiation—should adversely influence CNS development by mechanisms identified as subserving air pollutant effects.

## Materials and Methods

**Figure 1:**
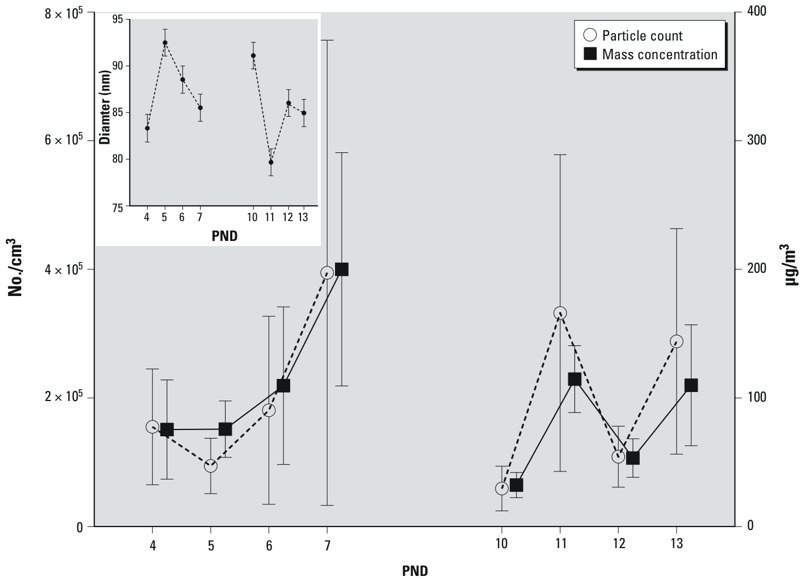
Mean particle counts and particle mass concentration for each day of exposure. Inset: mean diameter for each day of exposure. Error bars represent SDs.

*Animals, reagents, and exposures*. Eight-week-old male and female C57BL6/J mice were purchased from Jackson Laboratories (Bar Harbor, ME) and allowed to acclimate in the housing room for 1 week before breeding. Monogamous pairs of mice were bred for 3 days; males were then removed and dams remained singly housed with litters until weaning. Weanling mouse pups were exposed to ultrafine concentrated ambient particles (CAPs; < 100 nm) using the Harvard University Concentrated Ambient Particle System (HUCAPS) described previously (Allen et al. 2013). Briefly, animals were exposed to ambient UFPs in real time postnatal days (PNDs) 4–7 and on PNDs 10–13 for 4 hr/day for 4 days/week between 0700 and 1200 hours—the times corresponding to peak vehicular traffic outside the intake valve of the instrumentation. Particulates were concentrated approximately 10-fold the level in ambient outdoor air. The HUCAPS system is fitted with a size-selective inlet and a high-volume (5,000-L/min) UFP concentrator that concentrates ambient particles. CAPs- and filtered-air (FA)–treated animals received identical experimental manipulation. Because of the presence of the particle impactor in the HUCAPS system, animals in the CAPs-exposed chamber may have been held at a slightly higher negative pressure compared with animals treated with FA; however, the flow of CAPs-enriched or filtered air was maintained constant in both chambers. Room air was filtered by HEPA filtration (99.99% effective) for FA-exposed animals. Relative humidity and temperature in exposure chambers were maintained at 35–40% and 77–79°F (25–26°C). Particulate mass concentration and counts are shown in Figure 1. We obtained particle counts using a condensation particle counter (model 3022A; TSI, Shoreview, MN), and we calculated mass concentration using idealized particle density (1.5 g/cm^3^). On PND14 and PND55, the animals were euthanized by rapid decapitation to avoid the known effects of anesthetics on neurochemistry, allowing us to assess the immediate and persistent effects of CAPs exposure on the developing and young adult mouse CNS. An additional group of brains, from male mice euthanized at PND270 (from a separate exposure study), was examined for ventricle area. Exposure characteristics for the PND270 group were similar to those for the PND14 and PND55 groups. Details have been reported previously (Allen et al. 2014b). To preclude litter-specific effects, in the present study we used only one pup per sex per litter per time point. All mice used in this study were treated humanely and with regard for alleviation of suffering, and the study protocol was approved by the University of Rochester Institutional Animal Care and Use Committee.

*Glial fibrillary acid protein (GFAP) and ionized calcium-binding adapter molecule 1 (IBA-1) immunostaining and image analysis*. Brains were extracted and placed into 4% paraformaldehyde for 24 hr and then into 30% sucrose until they sank. The brains were sectioned on a freezing microtome (Microm HM 440 E; GMI Inc., Ramsey, MN) at 40-μm thickness in cryoprotectant (30% sucrose, 30% ethylene glycol in 0.1 M phosphate buffer) and stored at –4°F (–20°C) until immunostaining. Every sixth section was stained for GFAP and IBA-1 to assess the global activation of astrocytes and microglia, respectively. Briefly, the brain sections were washed of cryoprotectant and placed into primary antibody solutions for GFAP (AB5804, 1:4000 dilution; Millepore, Billerica, MA) or IBA-1 (016-20001, 1:5000; Wako Chemicals USA, Richmond, VA) for 24 hr. For GFAP, the tissue was then placed into a biotinylated secondary antibody solution (BA1000, 1:200 dilution; Vector Labs, Burlingame, CA) for 1 hr, and the stain was visualized using DAB (3-3´-diaminobenzidine). For IBA-1, the tissue was placed into a fluorescent-labeled secondary antibody solution (A-11012, 1:400 dilution; Life Technologies, Grand Island, NY). Immunolabeled tissue was mounted onto Superfrost Plus micro slides (48311-703; VWR, Radnor, PA) and coverslipped using Cytoseal 60 (for chromogenic tissue) (23-244257; Fisher Scientific, Pittsburg, PA) or ProLong Gold Antifade Reagent (P36930; Life Technologies).

Three images of each of following brain regions were obtained: corpus callosum, cortex, ventral midbrain, dentate gyrus, hippocampus (CA1/CA2), and striatum. Relative immunoreactivity was determined using Image Pro Plus version 7.0 (MediaCybernetics, Rockville, MD). All images underwent contrast enhancement before utilization of the count/size method. Briefly, immunoreactive cells on two or three sections per brain region were enumerated using the count/size feature of Image Pro Plus version 7.0 across three equally sized fields per brain region [method modified from Cao et al. (2012)]. Data are reported as percentages of time point– and sex-matched controls.

*Lateral ventricle and aqueduct of Sylvius area determination*. The area of the lateral ventricles (approximate bregma range: 1.10–0.38 mm) and aqueduct of Sylvius (approximate bregma range: –3.88 to –4.84 mm) was determined by tracing the outline of the area of interest in at least four adjacent sections of slide-mounted brain tissue using Neurolucida (MBF, Villiston, VT). We used Neurolucida software to enumerate the area of interest area in square micrometers. Lateral ventricle bregma for the PND14 brains are approximate, given that, to the knowledge of the authors, no atlas for that point in early postnatal brain development exists. To examine the persistence of lateral ventricle dilation in CAPs-exposed male mice, the ventricle area was quantified in brain tissue harvested at approximately PND270 from another group of identically, but not concurrently, exposed males. Unlike the mice from which brains were obtained at PND14 and PND55, mice from which PND270 tissue was harvested had undergone behavioral testing (reported by Allen et al. 2014b).

*Neurotransmitters quantification*. Briefly, brains were extracted and dissected, on an ice-cold plate, into the following regions: olfactory bulb, hippocampus, midbrain, striatum, hypothalamus, cerebellum, and cortex. We used HPLC coupled with an electrochemical detector (for monoamines) or a fluorescent detector (for amino acids) to quantify dopamine (DA), 3,4-dihydroxyphenylacetic acid (DOPAC), homovanillic acid (HVA), norepinephrine (NE), serotonin (5-HT), 5-hydroxyindoleacetic acid (5-HIAA), glutamine (GLN), glutamate (GLU), and γ-aminobutyric acid (GABA), expressed in nanograms per milligram protein. Dopamine turnover (DA TO) was calculated as DOPAC/DA concentrations. Method details are available elsewhere (Cory-Slechta et al. 2004, 2013; Virgolini et al. 2008).

*Cytokines*. Interleukin 1-beta (IL-1β), tumor necrosis factor-alpha (TNFα), and IL-6 in the striatum, hippocampus, olfactory bulb, midbrain, cortex, and cerebellum were quantified using custom multiplex plate–based chemiluminescent ELISA (Quansys Biosciences, Logan, UT). Briefly, tissue was quickly sonicated in 0.1 M PBS, pH 7.4, containing 1% protease inhibitor cocktail (P8340; Sigma, St. Louis, MO). Twenty-five microliters of brain homogenate was loaded per well and run in duplicate according to the manufacturer’s instructions. The chemiluminescent signal was visualized using the Q-view Imager and analyzed using Q-View Software (Quansys Biosciences). Cytokine levels were normalized to the total protein content of the same region as determined by the bicinchoninic acid method.

*Statistical analyses*. Statistical analysis was carried out using JMP10 (SAS Institute Inc., Cary, NC). Lateral ventricle dilation was characterized by the statistical interaction of postnatal CAPs × sex; thus, all statistical analyses were separated by sex and included two-way analyses of variance with age of sacrifice and treatment group as the independent factors. Fisher’s least significant difference post hoc analysis was used in the event of an age of sacrifice × treatment interaction. All analyses were performed as two-tailed tests, and *p* < 0.05 was considered statistically significant.

## Results

Mice were exposed on PNDs 4–7 and 10–13 for 4 hr/day, from 0700 to 1200 hours: the times corresponding to peak vehicular traffic and particle concentration levels near the intake valve. The mean CAPs count across the 8 days of postnatal exposure was approximately 200,000 particles/cm^3^, and the mean particle mass concentration was 96 μg/m^3^ (Figure 1). Particles remained in the UFP-size range for all exposure days (Figure 1 inset).

**Figure 2:**
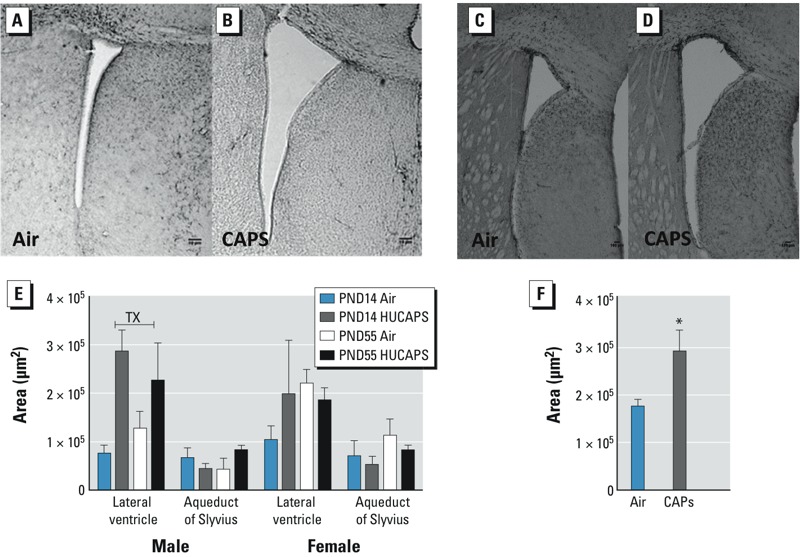
Images of lateral ventricle from air-exposed (*A,C*) and CAPS-exposed (*B,D*) male mice at PND14 (*A,B*) or at approximately PND270 (*C,D*). Bars = 10 μm. Quantification of lateral ventricle on PNDs 14 and 55 (*E*) and PND270 (*F*). The aqueduct of Sylvius area is shown for PNDs 14 and 55 (*E*). Data reported as group mean area ± SE. TX, main effect of CAPs treatment. *n* = 5 animals/sex/treatment/time point. *p < 0.05, by two-tailed *t*-test.

To address the potential of CAPs exposure to elicit immediate CNS effects and to determine the persistence of such effects, mice were euthanized at two time points after cessation of exposure: on PND14 (24 hr postexposure), and on PND55 (young adulthood). Notably, CAPs-exposed male, but not female, mice had significantly enlarged lateral ventricles compared with air-exposed controls (Figure 2A,B), as indicated by the treatment × sex interaction [*F*(1,32) = 10.0559, *p* < 0.01] and main effect of CAPs exposure for male mice [*F*(1,16) = 10.3298, *p* = 0.0054] and female mice [*F*(1,16) = 0.2455, *p* = 0.6270]. This observation was confirmed by Neurolucida (MBF Bioscience)–quantitated increases of lateral ventricle areas: 380% and 178% at PND14 and PND55, respectively (Figure 2E). On PND14 only, a single CAPs-treated female showed enlarged lateral ventricles as indicated by increased variability in Figure 2E for CAPs-females at that time point; however, this effect failed to reach statistical significance. Lateral ventricle size in mice (sex unspecified) has been reported to increase out to 90 days of age, with more rapid growth up to about 40 days of age (Mandell et al. 2010), although separate determinations by sex reveal that males show a decline in area between 1 and 3 months of age (Mandell et al. 2010). Figure 2A shows representative images of lateral ventricles of PND14 male air-treated (Figure 2A) and CAPs-treated (Figure 2B) animals at similar bregma. CAPs exposure did not affect the aqueduct of Sylvius (Figure 2E). To further evaluate persistence, brains from identically exposed males obtained at PND270 were subjected to ventricular tracing and increased lateral ventricle size was confirmed by a two-tailed *t*-test (*p* = 0.04; Figure 2C,D).

**Figure 3:**
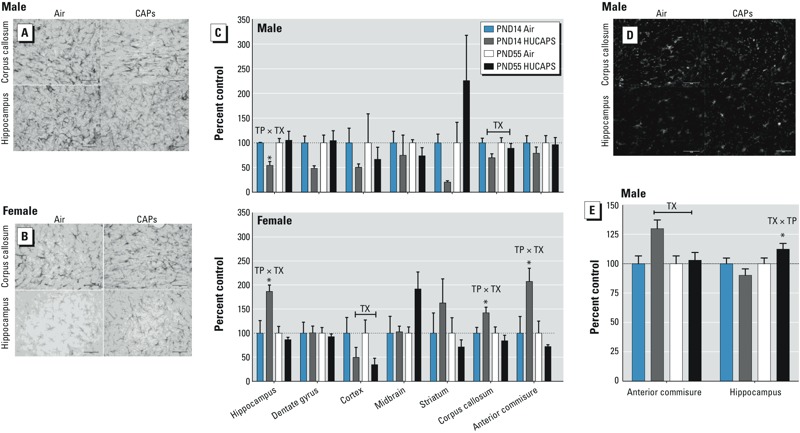
Representative images of GFAP immunoreactivity in the corpus callosum and hippocampus of air- and CAPs-exposed male (*A*) and female (*B*) mice at PND14, with (*C*) relative quantification of GFAP in those regions and in the dentate gyrus, cortex, midbrain, striatum, and anterior commissure immediately adjacent. (*D*) Representative images of IBA-1 immunoreactivity in the hippocampus and corpus callosum of male mice at PND55. (*E*) Relative quantification of IBA-1 immunoreactivity in the anterior commissure at PND14 and hippocampus of male mice at PND55. Data reported as percent sex-specific control by time point ± SE. Abbreviations: TX, main effect of CAPs treatment; TP × TX, statistical interaction between CAPs treatment and time point. Bar = 50 μm. *n* = 5 animals/sex/treatment /time point. *p < 0.05, compared with time point– and sex-specific control, by two-tailed *t*-test.

To assess glial activation as a potential mechanism of neurotoxicity in CAPs-exposed mice, we immunostained brain sections for GFAP and IBA-1, markers for astrocytes and microglia, respectively. CAPs exposure altered the astrocyte state in a sex- and regionally dependent manner (Figure 3A–C). Data are presented as percent time point– and sex-specific FA control values. In males, CAPs exposure reduced GFAP immunostaining in the corpus callosum [main effect of CAPs: *F*(1,15) = 4.5986, *p* = 0.048], at both PND14 and PND55, and reductions in GFAP immunoreactivity in the hippocampus occurred only at PND14 [time point × treatment interaction; *F*(1,15) = 5.200, *p* = 0.0376, *p* < 0.05] (Figure 3A,C). In contrast, females showed increases in GFAP in the hippocampus [CAPs by time point: *F*(1,16) = 9.1589, *p* = 0.008], corpus callosum [CAPs by time point: *F*(1,16) = 5.8066, *p* = 0.0284], and anterior commissure [CAPs by time point: *F*(1,16) = 12.76, *p* = 0.018] (Figure 3B,C) that were restricted to PND14 relative to PND14 air-treated females (all *p*-values < 0.05), but not at PND55, indicating a transient astrocytic response to CAPs exposure that was present on PND14 but resolved by PND55. Data were also normalized to sex-specific PND14 FA control to allow for the examination of the trajectory of GFAP changes across the study period (see Supplemental Material, Figure S1).

CAPs exposure altered IBA-1 immunostaining in the anterior commissure and hippocampus only in males (Figure 3D,E). Data are presented as percent time point- and sex-specific FA control. Despite the larger increase at PND14, statistical analyses indicated that CAPs exposure increased IBA-1 immunostaining (approximately 25%) in the anterior commissure at both time points [main effect of CAPs: *F*(1,15) = 5.75, *p* = 0.03], indicating persistent microglial response in the white matter. In contrast, CAPs exposure increased IBA-1 immunoreactivity in the hippocampus only at PND55 [CAPs by time point: *F*(1,15) = 4.8791, *p* = 0.043] relative to FA-treated controls (*p* < 0.05). Data were also normalized to sex-specific PND14 FA control to allow for examination of the trajectory of IBA-1 changes across the study period (see Supplemental Material, Figure S2).

CAPs exposure modified CNS neurotransmitter levels in a sex- and region-dependent manner. CAPs exposure increased hippocampal GLU [*F*(1,26) = 5.5383, *p* = 0.0246], midbrain DA TO [*F*(1,26) = 6.6590, *p* = 0.0159], and cortical DA TO [*F*(1,18) = 8.4456, *p* = 0.0106] in males at both time points (Table 1). CAPs exposure increased cortical NE in males only at PND55 [CAPs by time point: *F*(1,26) = 5.13, *p* = 0.03] (Table 2). No treatment-related differences in levels of CNS neurotransmitters were observed in the olfactory bulb or hypothalamus nor in levels of DA, DOPAC, HVA, 5-HT, or 5-HIAA in the midbrain, striatum, cortex, or hippocampus (data not shown).

**Table 1:** Neurochemical disruption and cytokine changes (group mean ± SE) in hippocampus, cortex, midbrain, and striatum of CAPs-exposed male mice.

Exposure	DA TO	NE	IL-1β	TNFα	IL-6	GLU
Hippocampus
PND14
Air	NA	NA	3.36 ± 0.77	NA	0.58 ± 0.15	41.57 ± 1.46
CAPs	NA	NA	2.39 ± 0.52	NA	0.35 ± 0.09	52.47 ± 4.80
PND55
Air	NA	NA	2.76 ± 0.16	NA	0.61 ± 0.05	52.27 ± 2.92
CAPs	NA	NA	2.09 ± 0.24	NA	0.45 ± 0.07	54.85 ± 3.03
Overall effects	NA	NA	TX	NA	TX	TX
Cortex
PND14
Air	22.35 ± 7.11	3.29 ± 0.17	0.97 ± 0.13	NA	NA	NA
CAPs	47.47 ± 21.16	3.29 ± 0.09	0.74 ± 0.06	NA	NA	NA
PND55
Air	3.43 ± 0.93	7.61 ± 0.31	2.71 ± 0.48	NA	NA	NA
CAPs	6.45 ± 1.57	8.82 ± 0.31*	1.70 ± 0.20	NA	NA	NA
Overall effects	TP, TX, TP × TX	TP, TX, TP × TX	TP, TX	NA	NA	NA
Midbrain
PND14
Air	4.26 ± 0.28	NA	20.33 ± 4.50	3.47 ± 0.76	NA	NA
CAPs	5.75 ± 0.62	NA	10.23 ± 0.911*	1.80 ± 0.15*	NA	NA
PND55
Air	1.01 ± 0.13	NA	19.99 ± 1.86	3.38 ± 0.29	NA	NA
CAPs	1.23 ± 0.20	NA	27.85 ± 6.710*	4.90 ± 1.12*	NA	NA
Overall effects	TP, TX	NA	TP, TP × TX	TP, TX × TX	NA	NA
Striatum
PND14
Air	NA	NA	2.11 ± 0.37	0.93 ± 0.23	NA	NA
CAPs	NA	NA	1.57 ± 0.24	0.52 ± 0.11	NA	NA
PND55
Air	NA	NA	1.39 ± 0.30	0.20 ± 0.04	NA	NA
CAPs	NA	NA	0.53 ± 0.11	0.10 ± 0.02	NA	NA
Overall effects	NA	NA	TP, TX	TX	NA	NA
Abbreviations: NA, not available; TP, statistical main effect of time point; TP × TX, statistical interaction; TX, statistical main effect of treatment. Monamines (DA TO, NE) reported in ng/mg protein, cytokines (IL-1β, IL-6, TNFα) reported in pg/mg protein. *n* = 8–12 animals/treatment/time point. *p < 0.05, statistically different from time point–specific air control.						

**Table 2:** Neurochemical disruption and cytokine changes (group mean ± SE) in hippocampus, cortex, midbrain, and striatum of CAPs-exposed female mice.

Exposure	DA TO	NE	DA	HVA	5-HT	IL-1β	TNFα	IL-6	GABA
Hippocampus
PND14
Air	72.26 ± 15.97	NA	NA	NA	8.65 ± 0.49	NA	NA	NA	3.68 ± 0.10
CAPs	99.94 ± 8.45*	NA	NA	NA	9.21 ± 0.53	NA	NA	NA	2.81 ± 0.35
PND55
Air	18.32 ± 5.68	NA	NA	NA	22.46 ± 1.34	NA	NA	NA	4.87 ± 0.45
CAPs	13.64 ± 2.44	NA	NA	NA	28.30 ± 1.33*	NA	NA	NA	4.23 ± 0.32
Overall effects	TP, TP × TX	NA	NA	NA	TP, TX, TP × TX	NA	NA	NA	TP, TX
Cortex
PND14
Air	NA	3.46 ± 0.24	NA	NA	NA	NA	NA	< LOD	NA
CAPs	NA	3.54 ± 0.13	NA	NA	NA	NA	NA	< LOD	NA
PND55
Air	NA	8.27 ± 0.23	NA	NA	NA	NA	NA	0.36 ± 0.08	NA
CAPs	NA	7.25 ± 0.201*	NA	NA	NA	NA	NA	0.18 ± 0.028*	NA
Overall effects	NA	TP, TX, TP × TX	NA	NA	NA	NA	NA	NA	NA
Midbrain
PND14
Air	5.85 ± 0.69	NA	1.83 ± 0.17	5.40 ± 0.33	NA	11.50 ± 2.07	2.13 ± 0.32	1.55 ± 0.31	NA
CAPs	3.83 ± 0.40*	NA	2.81 ± 0.25	6.51 ± 0.39	NA	13.27 ± 1.89	2.39 ± 0.24	1.56 ± 0.23	NA
PND55
Air	1.34 ± 0.11	NA	2.19 ± 0.18	2.30 ± 0.16	NA	14.95 ± 1.45	2.72 ± 0.24	2.34 ± 0.26	NA
CAPs	1.29 ± 0.08	NA	2.37 ± 0.21	2.28 ± 0.13	NA	27.72 ± 3.12	4.77 ± 0.49	4.84 ± 0.588*	NA
Overall effects	TP, TX, TP × TX	NA	TX	TP, TX	NA	TX	TP, TX	TP, TX, TP × TX	NA
Striatum
PND14
Air	NA	NA	NA	NA	NA	NA	0.19 ± 0.02	0.40 ± 0.14	NA
CAPs	NA	NA	NA	NA	NA	NA	0.13 ± 0.019*	0.15 ± 0.034*	NA
PND55
Air	NA	NA	NA	NA	NA	NA	0.11 ± 0.02	0.05 ± 0.01	NA
CAPs	NA	NA	NA	NA	NA	NA	0.12 ± 0.02	0.07 ± 0.01	NA
Overall effects	NA	NA	NA	NA	NA	NA	TP, TX, TP × TX	TP, TX, TP × TX	NA
Abbreviations: LOD, limit of detection; NA, not available; TP, statistical main effect of time point; TP × TX, statistical interaction; TX, statistical main effect of treatment. Monamines (DA TO, NE, DA, HVA, and 5-HT) reported in ng/mg protein, amino acid (GABA) reported in μg/mg protein, cytokines (IL-1β, IL-6, TNFα) reported in pg/mg protein. *n* = 8–12 animals/treatment/time point. *p < 0.05, statistically different from time point–specific air control.									

In females, CAPs exposure reduced hippocampal GABA [*F*(1,28) = 4.22, *p* = 0.049], but increased midbrain HVA [*F*(1,29) = 4.92, *p* = 0.035] and DA [*F*(1,29) = 6.9, *p* = 0.013] and hippocampal 5-HT [*F*(1,29) = 6.46, *p* = 0.017] at both time points (Table 2). In addition, cortical NE was increased only at PND55 [CAPs by time point: *F*(1,29) = 6.37, *p* = 0.017], whereas hippocampal DA TO [CAPs by time point: *F*(1,26) = 4.90, *p* = 0.036] was increased, and midbrain DA TO was reduced [CAPs by time point: *F*(1,29) = 9.52, *p* = 0.004], but only at PND14. No treatment-related differences in neurotransmitter levels in the olfactory bulb, cerebellum, or hypothalamus were observed (data not shown).

In males, reductions in hippocampal IL-6 [*F*(1,28) = 4.69, *p* = 0.039], and in striatal IL-1β [*F*(,1,28) = 6.48, *p* = 0.017] and TNFα [*F*(1,28) = 5.00, *p* = 0.033], were observed at both time points, with a similar trend in hippocampal IL-1β [*F*(1,28) = 3.59, *p* = 0.069] (Table 1). Hippocampal GLU in CAPs-exposed males was positively correlated with hippocampal IL-1β (*R*^2^ = 0.233, *p* = 0.039) and IL-6 (*R*^2^ = 0.361, *p* = 0.01). In males, midbrain IL-1β [CAPs by time point: *F*(1,28) = 4.76, *p* = 0.038] and TNFα [CAPs by time point: *F*(1,28) = 5.448, *p* = 0.027] were reduced at PND14, but increased at PND55 (all *p*-values < 0.05) (Table 1). We observed no treatment-related differences in central cytokines in the male olfactory bulb or cerebellum (data not shown).

In CAPs-exposed females at PND55, cortical IL-6 was reduced [CAPs: *F*(1,18) = 5.78, *p* = 0.027] but midbrain IL-6 was increased [CAPs by time point: *F*(1,28) = 5.92, *p* = 0.022] (Table 2). We detected no IL-6 in the female cortex at PND14. Midbrain TNFα [*F*(1,28) = 7.05, *p* = 0.013] and IL-1β [*F*(1,28) = 6.65, *p* = 0.016] were increased in CAPs-exposed females at both time points (Table 2). In contrast, striatal IL-6 was decreased at PND14, but not at PND55 [CAPs by time point: *F*(1,28) = 8.61, *p* = 0.007, all post hoc *p* values < 0.05] (Table 2). No treatment-related differences in cytokine concentrations were observed in the olfactory bulb or cerebellum (data not shown).

## Discussion

We exposed mice to human-relevant levels of UFP. As indicated in Figure 1, the average particle count was approximately 200,000 particles/cm^3^. Ambient UFP counts near roadways in Los Angeles, California (Westerdahl et al. 2005) and Minneapolis, Minnesota (Kittelson 2004) have been reported to be as high as 200,000 and 400,000 particles/cm^3^, respectively, with peak episodic counts reaching 2,000,000 particles/cm^3^ in Minneapolis (Kittelson 2004).

We observed a persistent dilation of the lateral ventricles, but not of the aqueduct of Sylvius, that occurred preferentially in CAPs-exposed male mice. Lateral ventricle dilation is a predictor of poor neurodevelopmental outcome (Laskin et al. 2005; Tatli et al. 2012). It has been associated with multiple developmental CNS disorders, including autism and schizophrenia (Barttfeld et al. 2011; Bigler 1987; Fannon et al. 2000; Movsas et al. 2013; Sanfilipo et al. 2000; Schulz et al. 1983; Wright et al. 2000), idiopathic mental retardation, periventricular leukomalacia (Volpe 2001, 2003, 2005), fragile X syndrome, and attention deficit disorder and, in the absence of other CNS abnormalities, to developmental delays (Gilmore et al. 2001, 2008). Its consequences can include progressive hydrocephalus, gray matter migration abnormalities, loss of parenchymal brain tissue, agenesis of the corpus callosum, and delayed or abnormal maturation of white matter, that is, reduced MBP (myelin basic protein) expression, diminished total axon volume, trisomies, and microcephaly (Bigler 1987; Gilmore et al. 1998, 2001, 2008; Griffiths et al. 2010; Kuban et al. 1999; Kyriakopoulou et al. 2014, Manfredi et al. 2010). Ventriculomegaly is associated with such deficits, persists after birth (Gilmore et al. 2001), and is more prevalent in males (Gilmore et al. 1998). Our observation of male specificity of the lateral ventricle dilation is consistent with literature suggesting that males are more likely to be diagnosed with a number of neurodevelopmental and neuropsychological disorders of childhood, including autism, earlier onset schizophrenia, attention deficit hyperactivity disorder, conduct disorder, and learning disabilities (Boyle et al. 2011; Centers for Disease Control and Prevention 2007; Erskine et al. 2014; Kirkbride et al. 2012). Although the mechanism(s) underlying the male specificity of this effect are as yet undefined, they likely reflect sex differences in neurodevelopment such as the sex differences in microglial colonization of the brain already seen by PND4 [at which time males show a more activated morphology (Schwarz et al. 2012)], a possibility that is consistent with the observation that changes in IBA-1 were found only in males. Although we observed a single CAPs-exposed female that appeared to have enlarged lateral ventricles at PND14, the effects were not statistically significant, further suggesting male specificity of the lateral ventricle dilation. However, future studies should address whether females may be rendered susceptible at higher particle concentrations or at longer durations of exposure. Obstruction of the aqueduct of Sylvius is a common mechanism of lateral ventricle dilation (James 1992), but that was not seen in the present study. Whether an earlier transient obstruction occurred cannot be ruled out, however. Future studies of a similar nature should include the use of a repeated-measures design in rodents exposed to CAPs. Use of magnetic resonance imaging to track the trajectory of central ventricular system changes in the same animal across time would assist in illuminating the mechanism(s) by which such changes occur in CAPs-exposed male mice and may inform the sex dependency of this effect. Global patterns of glial changes in the brain indicate that females mount a transient astroctyic response—but no microglial response—to CAPs exposure, whereas males show both astrocytic and microglial dysfunction that persists into early adulthood.

Our previous work indicates that significant disruption of adulthood neurotransmission in response to CAPs exposure in mice persists at least to almost 1 year of age (Allen et al. 2014a, 2014b). To determine the etiological role of such disruption in CAPs-associated neuropathology, we examined regional levels of DA and its metabolites, DOPAC, HVA, NE, 5-HT and its metabolite 5-HIAA, as well as GLN, GLU, and GABA. Notably, the sustained increase in hippocampal GLU may indicate the contribution of an excitotoxic mechanism of CAPs that persists until early adulthood. In addition, increased DA metabolism, as evidenced in our animals by increased DA TO, has been associated with oxidative stress (Cohen 1983; Graham 1978; Hastings 1995; Schulz et al. 2000). Interestingly, a loss of GABAergic neurons in the hippocampus, which is consistent with the decreased hippocampal GABA observed in females in the present study, has been implicated in both schizophrenia and bipolar disorder (Benes et al. 1998). Moreover, disrupted CNS neurotransmission is associated with both autism (Cook 1990) and schizophrenia (Grace 2012).

Early cytokine changes were also sex and brain-region dependent (Tables 1 and 2). In the female midbrain, IL-6 was increased only at PND55, whereas TNFα and IL-1β were persistently increased across both time points. IL-6 in the female striatum was increased only at PND14. A protracted profile of changes, as observed for female midbrain IL-6, may indicate adverse effects on the ontogeny of microglial development that later results in a neuroinflammatory profile; whereas changes that were restricted to PND14 only, such as was observed for female striatal IL-6, likely indicate a transient response.

Decreases, as opposed to increases, in male hippocampal IL-6 and striatal IL-1β/TNFα and in female cortical IL-6 were unanticipated, but perhaps suggest that microglia, a major source of brain cytokines, are dysfunctional or lost. Brain cytokines have multiple important roles in the developing nervous system (Deverman and Patterson 2009), such that any alteration in their levels during the early postnatal period would have deleterious effects on the CNS. Indeed, IL-1β, TNFα, and IL-6 have been implicated as having roles in synaptic plasticity in the hippocampus (Balschun et al. 2004; Goshen and Yirmiya 2009; Schneider et al. 1998) and can activate astrocytes that modulate synaptic plasticity. Furthermore, IL-1 receptor antagonist polymorphism has been implicated in ADHD (attention deficit hyperactivity disorder) etiopathogenesis (Segman et al. 2002), and disrupted attention has been observed previously in our male CAPs-exposed mice (Allen et al. 2013). Furthermore, hippocampal GLU in CAPs-exposed males was positively correlated with hippocampal IL-1β/IL-6, likely indicating a mechanistic link between excitotoxicity and neuroinflammatory responses. Fogal and Hewett (2008) have proposed IL-1β as a bridge between neuroinflammation and excitotoxicity. This correlation was absent in air-exposed control males and in females regardless of exposure group.

Collectively these data show a dramatic susceptibility of male mice to environmentally relevant levels of early postnatal air pollution exposure, with effects that persist into adulthood and cause permanent neuropathology characterized by ventricular enlargement, a pathology not seen in females. Lateral ventricle dilation (ventriculomegaly), is a strong predictor of poor neurodevelopmental outcome in children and a pathological hallmark observed in both autism and schizophrenia. Thus, the findings we present here provide biological plausibility for the reported associations in epidemiological studies of air pollution with autism (Becerra et al. 2013; Volk et al. 2011, 2013), schizophrenia (Pedersen et al. 2004), and ADHD (Siddique et al. 2011). Moreover, the heightened sensitivity of males to CAPs effects parallels the greater prevalence of these disorders in males.

Although ventricular enlargement is not observed in female mice exposed to CAPs, CAPs-exposed females exhibit biochemical and neurochemical alterations that are nevertheless significant and represent protracted neurotoxicity in response to early postnatal CAPs exposure. CAPs-exposed males and females show significantly altered neurochemical changes in multiple brain regions, including areas that comprise the mesocorticolimbic dopamine tracts, regions critical to cognition and attention. CAPs-exposed males have increased levels of the major excitatory neurotransmitter GLU in the hippocampus, a sign of excitotoxicity in that region. CAPs-exposed females show a decrease in hippocampal GABA, the major inhibitory neurotransmitter of the CNS. GABA alterations in the hippocampus have been implicated in both schizophrenia and bipolar disorder (Benes and Berretta 2001). The functional/behavioral significance of these changes remains to be fully determined; however, impairment in behaviors involving the hippocampus, such as learning and memory, would be predicted. These changes in neurotransmitters along the mesocorticolimbic pathway may underlie the increased preference for immediate reward observed in CAPs-exposed males that we previously reported (Allen et al. 2013). In interpreting these findings, inherent differences between murine and human brain development must also be considered. The early postnatal period in both humans and rodents is marked by substantial brain development; however, the exact nature of the development is different. As a rough estimate, rat brain development at PND7 has been determined to approximate human brain development at birth (Clancy et al. 2007); thus our paradigm for exposure from PND 4–7 and 10–13 in mice, in terms of neurodevelopment, probably best approximates what would be the perinatal period in the human, encompassing the time frame shortly before and after birth. Taken together, these data suggest that exposure to CAPs in the early postnatal period, at human- and environmentally relevant levels, may represent a far greater public health concern than has previously been recognized as a risk factor contributing to intractable neurodevelopmental disorders such as autism and schizophrenia.

## Supplemental Material

(700 KB) PDF
