# Supplementary material for: Early Postnatal Exposure to Ultrafine Particulate Matter Air Pollution: Persistent Ventriculomegaly, Neurochemical Disruption, and Glial Activation Preferentially in Male Mice
Source: Environ Health Perspect. 2014 Jun 5;122(9):939–45. doi: 10.1289/ehp.1307984 (PMC4154219; doi:10.1289/ehp.1307984)

Environmental Health Perspectives doi:10.1289/ehp.1307984

Supplemental Material

Early Postnatal Exposure to Ultrafine Particulate Matter Air Pollution: Persistent Ventriculomegaly, Neurochemical Disruption, and Glial Activation Preferentially in Male Mice

Joshua L. Allen, Xiufang Liu, Sean Pelkowski, Brian Palmer, Katherine Conrad, Günter Oberdörster, Douglas Weston, Margot Mayer-Pröschel, and Deborah A. Cory-Slechta

Figure S1. Relative quantitation of glial fibrillary acidic protein (GFAP) immunoreactivity in Air- and CAPS-exposed males and females in the hippocampus, dentate gyrus, cortex, midbrain, striatum, corpus callosum, and anterior commissure. Data reported as percent PND14 sex-specific control by time point ±SE. (n=5 animals/sex/treatment/time point). TP indicates main effect of time point, TX indicates main effect of CAPS treatment, TP x TX indicates their statistical interaction.

* indicates statistically different ($p<0.05$, two-tailed) from time point-specific control.

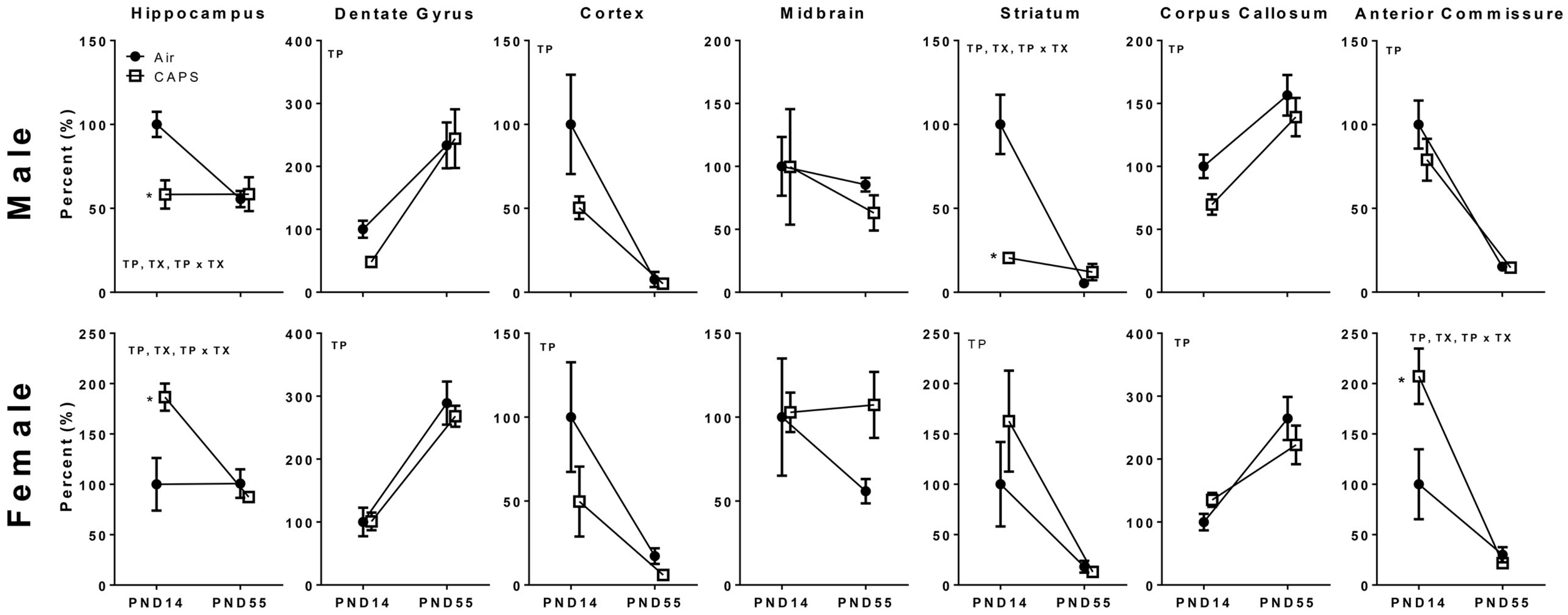

Figure S2. Relative quantitation of ionized calcium-binding adaptor molecule 1 (IBA-1) immunoreactivity in Air- and CAPS-exposed males in the anterior commissure and hippocampus. Data reported as percent PND14 sex-specific control by time point ±SE. (n=5 animals/sex/treatment/time point). TP indicates main effect of time point, TX indicates main effect of CAPS treatment, TP x TX indicates their statistical interaction.
* Indicates statistically different ($p<0.05$, two-tailed) from time point-specific control.

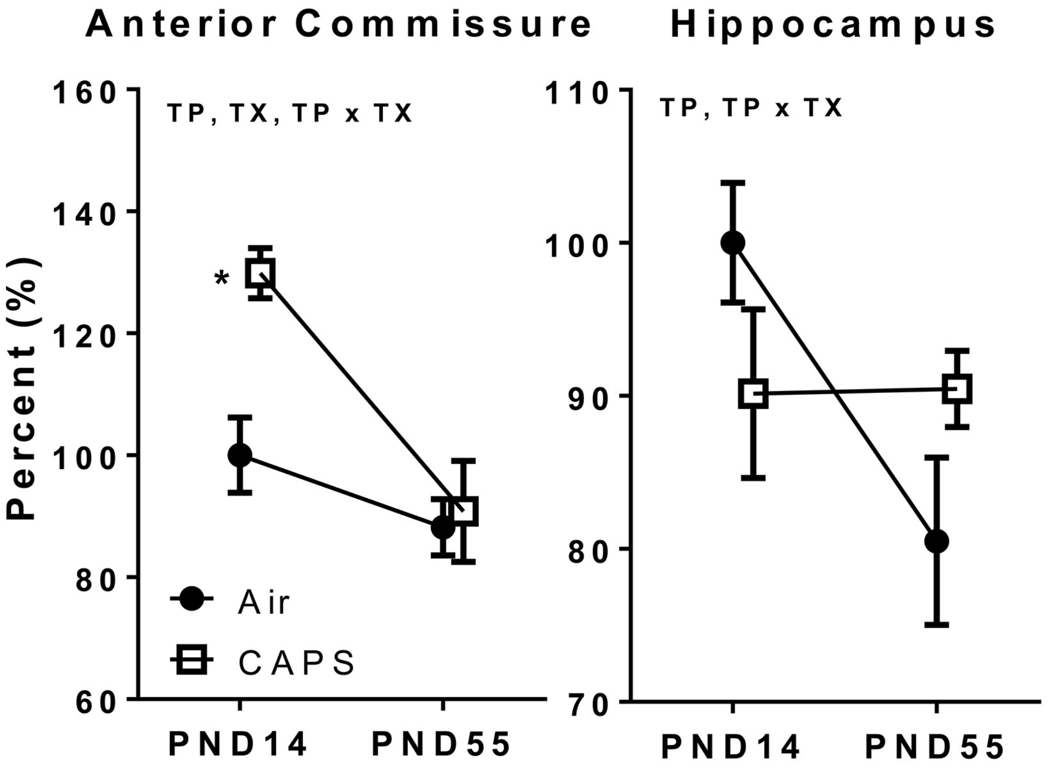

Supplement: (700 KB) PDF [file ehp.1307984.s001.pdf]
